# Evaluation of Phase-Amplitude Coupling in Resting State Magnetoencephalographic Signals: Effect of Surrogates and Evaluation Approach

**DOI:** 10.3389/fncom.2016.00120

**Published:** 2016-11-25

**Authors:** Bakul Gohel, Sanghyun Lim, Min-Young Kim, Kyung-min An, Ji-Eun Kim, Hyukchan Kwon, Kiwoong Kim

**Affiliations:** Center for Biosignals, Korea Research Institute of Standards and ScienceDaejeon, South Korea

**Keywords:** cross-frequency coupling, phase-amplitude coupling, resting state, magnetoencephalography

## Abstract

Phase-amplitude coupling (PAC) plays an important role in neural communication and computation. Interestingly, recent studies have indicated the presence of ubiquitous PAC phenomenon even during the resting state. Despite the importance of PAC phenomenon, estimation of significant physiological PAC is challenging because of the lack of appropriate surrogate measures to control false positives caused by non-physiological PAC. Therefore, in the present study, we evaluated PAC phenomenon during resting-state magnetoencephalography (MEG) signal and considered various surrogate measures and computational approaches widely used in the literature in addition to proposing new ones. We evaluated PAC phenomenon over the entire length of the MEG signal and for multiple shorter time segments. The results indicate that the extent of PAC phenomenon mainly depends on the surrogate measures and PAC computational methods used, as well as the evaluation approach. After a careful and critical evaluation, we found that resting-state MEG signals failed to exhibit ubiquitous PAC phenomenon, contrary to what has been suggested previously.

## Introduction

Cross-frequency coupling between the frequency components of neural oscillatory signals has incited considerable interest amongst the neuroscientists community (Canolty and Knight, 2010; Jirsa and Müller, 2013; Aru et al., 2015). Cross-frequency coupling, particularly phase-amplitude coupling (PAC), where the amplitude of a high frequency component is modulated with the phase of a low frequency component, has been claimed to play an important role in neural communication and neural information processing (Canolty and Knight, 2010; Voytek et al., 2013; Lega et al., 2016). It is believed that nested high-frequency neural oscillations reflect local cortical processing, whereas low-frequency neural oscillations are associated with global neuronal communication in response to external or internal events. Previously, PAC phenomenon was primarily evaluated for certain cognitive or physiological tasks or disease states (Yanagisawa et al., 2012; de Hemptinne et al., 2013; Voytek et al., 2013; Lega et al., 2016; van Wijk et al., 2016). PAC phenomenon may play a major role in the resting brain and in communication among resting-state networks. Recently, an MEG study on PAC during the resting state showed that 41–61% of cortical voxels exhibited significant PAC (Florin and Baillet, 2015). Recent invasive (electrocorticogram [EcoG] and local field potential [LFP]) studies have also suggested ubiquitous PAC phenomenon during the resting state that assists high-frequency communication across remote neuronal assemblies even in the absence of an overt task structure (Wang et al., 2012; Weaver et al., 2016).

Despite the importance of PAC phenomenon, estimation of a true physiological PAC is not straightforward. Previous studies have indicated that any abrupt change or imperfect sinusoids in the signal can lead to spurious PAC estimation (Kramer et al., 2008). Thus, non-physiological PAC can be observed for any signal even in the absence of neurophysiological PAC (Aru et al., 2015). For example, PAC phenomenon can be detected in atmospheric noise signals. In practice, one often has to rely on a surrogate measure to derive the statistical significance of the computed PAC index. Therefore, the extent of PAC also depends on the surrogate measure used for evaluation. Essentially, none of the surrogate measures are ideal, but some are more conservative than others (Aru et al., 2015). Recent opinions suggest that good surrogate data, particularly for a longer duration, can be generated through splitting either the phase or the amplitude time series components into two blocks at random times and then interchanging the positions of the blocks (Aru et al., 2015). This process will destroy the specific PAC, but will have a minimal detrimental effect on the cyclostationarity of the phase or amplitude components. Moreover, across the studies, a wide range of computational methods, such as modulation index (Özkurt and Schnitzler, 2011; Florin and Baillet, 2015), general linear model (Penny et al., 2008; Özkurt and Schnitzler, 2011; van Wijk et al., 2015), and oscillation-triggered coupling based PAC analysis (Dvorak and Fenton, 2014) have been used to capture the PAC phenomenon. Calculating Kullback–Leibler divergence between the phase-amplitude coupled distribution and the uniform distribution (Tort et al., 2010), and correlation-based PAC (Bruns and Eckhorn, 2004; Penny et al., 2008) have also been used to capture this phenomenon.

Thus, the objective of the present study was to evaluate PAC phenomenon in the resting-state MEG source signal considering various surrogate measures and PAC computation approaches and to integrate the cognizance of these into our conclusions. In the present study, we considered five different kinds of surrogate measures and three different PAC computational approaches, considering various aspects of the elementary raw input signals and PAC phenomenon.

## Materials and methods

### Dataset and preprocessing

**Figure 1:**
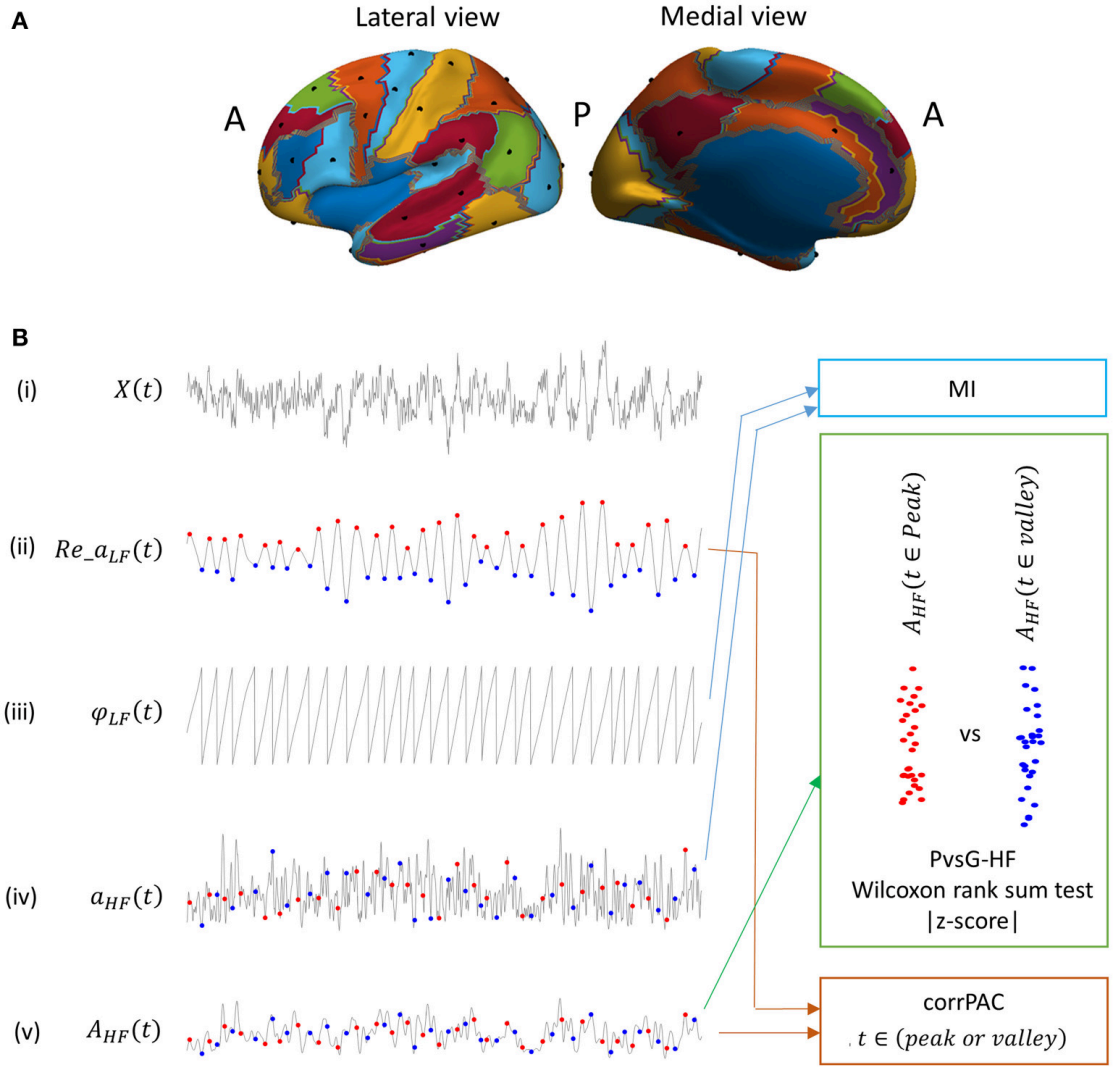
**(A)** Locations of nodes (black dots, 29 nodes) distributed over the cortex (left hemisphere) that were used for the analysis. Each colored partition represents one or more groups of Brodmann areas **(B)** Outline of low and high frequency components of the signal that were used for computing PAC score using various computational methods (MI, modulation index; PvsV-HF, peak vs. valley high frequency amplitude; and CorrPAC, correlation-based PAC). (i) Raw elementary source time series, (ii) real component of low frequency complex time series (low frequency component), (iii) phase of the low frequency component, (iv) high frequency amplitude time series (high frequency component), (v) high frequency amplitude time series smoothened with a Gaussian window function. Peak location (red dot) and Valley locations (blue dot) in a low frequency component.

Data used in the preparation of this work were obtained from the MGH-USC Human Connectome Project (HCP) database (https://ida.loni.usc.edu/login.jsp). The HCP project (Principal Investigators: Bruce Rosen, M.D., Ph.D., Martinos Center at Massachusetts General Hospital; Arthur W. Toga, Ph.D., University of California, Los Angeles, Van J. Weeden, MD, Martinos Center at Massachusetts General Hospital) is supported by the National Institute of Dental and Craniofacial Research (NIDCR), the National Institute of Mental Health (NIMH), and the National Institute of Neurological Disorders and Stroke (NINDS). Collectively, the HCP is the result of efforts of co-investigators from the University of California, Los Angeles, Martinos Center for Biomedical Imaging at Massachusetts General Hospital (MGH), Washington University, and the University of Minnesota (Larson-Prior et al., 2013). The preprocessed and cleaned resting state MEG datasets of 27 unrelated subjects were used in the present study. Originally, the MEG HCP database contained resting-state datasets from 61 subjects. However, we only chose 27 subjects among them for whom data are available from a resting-state session with continuous data with a length >4 min without any bad segments to avoid discontinuities in data. Moreover, at least 10 independent components (ICs) were left out after independent component analysis (ICA)-based artifactual component pruning (for subject and session details, refer to the Supplementary Text A). The detailed experimental set-up and data-preprocessing pipeline are available at (http://www.humanconnectome.org/documentation/S500/HCP_S500+MEG2_Release_Reference_Manual.pdf). In brief, eye open resting-state MEG data were acquired in three sessions, and each session was 5 min long. MEG data were recorded using the whole-head MGNES 3600 (4D Neuroimaging, San Diego, CA) system with 248 magnetometer channels at a sampling rate of 2034.51 Hz. The MEG signals were filtered using a band-pass (1.3–150 Hz) and a notch (59–61/119–121 Hz) Butterworth filter and then downsampled to 508.675 Hz. ECG- and EOG-related artifact data were removed using ICA-based approach. Anatomical MRI was recorded for each subject. A single-shell volume conduction model was used for the head model, and a cortical sheet with 8004 vertices defined on the normalized space was used for the source model. An elementary source signal for each of the vertices was reconstructed from the sensor signal using the weighted minimum norm estimate (wMNE) inverse solution (Hämäläinen et al., 1993). We used the publicly available FieldTrip library for the computation of the inverse solution (Oostenveld et al., 2011). During the computation of the inverse solution, a channel covariance matrix was computed using a 5 min empty-room MEG signal, whereas a regularization parameter (that controls the magnification of error related to signal-to-noise ratio) and a depth-weighting factor were set to default values of 3 and 0.5, respectively. We divided the cortex into 46 partitions based on Brodmann areas or groups of Brodmann areas (Figure 1A) and chose one or two nodes from each of the partitions. Thus, in the present study, we used a total of 58 nodes distributed over the cortex (as shown in Figure 1A) for analysis. For more detail, please refer to the Supplementary Text B.

### Surrogate data generation

As there is no direct measure available to establish the significance of an estimated PAC score, one often has to rely on surrogate data, i.e., the bootstrapping approach (Özkurt and Schnitzler, 2011; Dvorak and Fenton, 2014; Aru et al., 2015). In the present study, we generated various kinds of surrogate data having a null hypothesis of no neurophysiological PAC phenomenon. The first kind of surrogate data was produced by splitting either phase or amplitude time series into two blocks at some random time point and then interchanging their positions. This process destroys the specific phase-amplitude relationship but only minimally distorts the cyclostationarity of the low-frequency (LF) and high-frequency (HF) components (Aru et al., 2015). We refer to this as the “PhaseRand” surrogate measure in the subsequent text. Another kind of surrogate dataset was generated by permuting the data of the elementary source signal. We refer to this as the “RandPerm” surrogate measure in the subsequent text. These surrogate random time series have distributions identical to the elementary source signal. Another kind of surrogate dataset was generated by simply considering Gaussian time series with similar duration to that of the original time series. We refer to this as the “Gaussian” surrogate measure in the subsequent text. Moreover, the aforementioned RandPerm and Gaussian surrogate data lack the 1/*f* signal characteristics of the elementary source signal. Therefore, another kind of surrogate data was generated by coloring (introducing 1/*f* characteristics) the data in such a way that they have spectral characteristics similar to the elementary source signal. For the coloring, we computed Fourier transforms of the surrogate signal and the elementary source signal. Then, the amplitude of each Fourier coefficient of the surrogate signal was set as the amplitude of the corresponding Fourier coefficient of the elementary source signal without changing phase information. Then, the inverse Fourier transform was applied to produce a surrogate signal with 1/*f* spectral characteristics. In total, we employed five different kinds of surrogate measures to evaluate resting state PAC phenomenon, which are “PhaseRand,” “RandPerm(c–),” “RandPerm(c+),” “Gaussian (c–),” and “Gaussian(c+).” Here, c– and c+ indicate without applying coloring and with applying coloring to the surrogate data, respectively.

### PAC score computation

PAC implies fluctuation of the HF amplitude in association with the phase (peaks and valleys) of the LF component of the signal. Prerequisite to computing the PAC score, the phase and/or amplitude of the LF component and the amplitude of the HF component need to be extracted from the elementary source signal. Earlier studies primarily used either the Hilbert envelope-based method or a complex wavelet transform to extract the LF and HF components from the signal (Penny et al., 2008; Dvorak and Fenton, 2014; Aru et al., 2015). In the present study, the LF and HF components were extracted through a complex Morlet wavelet with a time resolution of 1 s (i.e., FWHM = 1) and a central frequency of 1 Hz (i.e., *f*_c_ = 1 Hz). For this, we used the publicly available Brainstorm Software library for the MATLAB programming platform (Tadel et al., 2011). The choice of the frequency value for the LF and HF components differs across studies. A recent study on resting-state PAC based on MEG signals showed significant PAC primarily in the delta, theta, and alpha LF bands (Florin and Baillet, 2015). Therefore, we evaluated PAC for low frequencies [i.e., 3 Hz(δ, delta), 6 Hz(θ, theta) and 10 Hz(α, alpha)]. The choice of the high frequency value also widely varies across the studies, but it is often chosen to be above 80 Hz and evaluated using the LFP or the EcoG signal (Canolty et al., 2006; Voytek et al., 2013; van Wijk et al., 2016). However, MEG has a limited signal-to-noise ratio particularly for high frequency oscillations (Muthukumaraswamy, 2013). We evaluated PAC for two HF bands [53–93 Hz (γ_*L*_, lower gamma) and 83–143 Hz (γ_*H*_, higher gamma)]. Thereby, in total, we evaluated PAC separately for six different frequency pairs— (α−γ_*L*_), (α−γ_*H*_), (θ−γ_*L*_), (θ−γ_*H*_), (δ−γ_*L*_), and (δ−γ_*H*_). Frequency resolution is lower at higher frequencies for the Morlet wavelet transform. Therefore, the amplitude time course for HF components was first computed for the individual frequency in 10 Hz steps (for γ_*L*_ = 53:10:93 Hz and for γ_*H*_ = 83:10:143 Hz) and then summed to obtain the single HF amplitude time course for lower gamma (γ_*L*_) and higher gamma (γ_*H*_) bands (Equation 2).

(1) $$\begin{array}{r} C\;\left(\omega ,t\right)=\text{complex morlet transform}\left(X\left(t\right),\;\omega \right) \end{array}$$

where ω is the frequency and *X*(*t*) is the elementary raw signal.

(2) $$\begin{array}{rcl} a_{\text{HF}}\left(t\right) & = & \sum_{\omega \;\epsilon \text{ HF}}|C\left(\omega ,t\right)| \end{array}$$

(3) $$\begin{array}{rcl} \varphi_{\text{LF}}\left(t\right) & = & \text{angle}\left(C\left(\omega ,t\right)\right),\;\omega \in \text{LF} \end{array}$$

(4) $$\begin{array}{rcl} \text{Re}\_a_{\text{LF}}\left(t\right) & = & \text{real}\left(C\left(\omega ,t\right)\right),\;\omega \in \text{LF} \end{array}$$

As discussed above, a wide range of PAC computation methods are available in the literature, which find association between the phase of the LF component and the amplitude of the HF component. However, in the present study, we used the following three different PAC computational approaches.

#### PAC based on modulation index (MI)

Modulation index (MI) is a direct computational approach for PAC scoring that has been frequently used in various studies (Özkurt and Schnitzler, 2011; Florin and Baillet, 2015). In the present study, we also used it as one of the PAC score computation approaches (Equation 5). Higher MI-values indicate a greater association between the phase of LF components and the amplitude of HF components.

(5) $$\begin{array}{r} \text{MI }=\frac{1}{N}\frac{\left|\sum_{t=1}^{N}a_{\text{HF}}\left(t\right){\text{e}}^{\text{i}\varphi_{\text{LF}}\left(t\right)}\right|}{\sqrt{\sum_{t}a_{\text{HF}}{\left(t\right)}^{2}}} \end{array}$$

Where φ_LF_ represents the LF phase time course, *a*_HF_(*t*) represents the HF amplitude time course and *N* is the length of the data.

#### PAC based on peak vs. valley HF amplitude (PvsV-HF)

In PAC, the amplitude of the HF component is locked to either the peaks or the valleys of the LF component (because of ±180° ambiguity of power in the MEG signal). Consequently, if PAC is present, the distribution of the HF amplitude at the peaks should be higher compared to at the valleys or vice versa. Therefore, PAC phenomenon can be captured by a direct statistical comparison of the HF amplitude at the peaks of LF component against the HF amplitude at the valleys of LF component (Figure 1B). First, we identified the peak and valley by finding the local maxima and minima in the LF amplitude time course (Re_*a*_LF_(*t*)), and the corresponding phase value, i.e., 0° ± 15° For the peak and 180° ± 15° for the valley in the LF phase time course (φ_LF_(*t*)). The HF amplitude time course (*a*_HF_(*t*)) was smoothed by convolving it with a Gaussian window function that had a length one-fourth of a single cycle of the LF-value in a frequency pair (*A*_HF_, Equation 6). Then, the amplitudes at each of the peaks [red dot, Figure 1B(v)] and valleys [blue dot, Figure 1B(v)] in this smoothed HF amplitude time course (*A*_HF_) were extracted. In other words, we extracted weighted mean HF amplitudes at the peak or valley locations considering a time segment of ±45° around the peak or valley time location. Thereafter, distribution of the HF amplitude at peak locations was statistically compared against the HF amplitude at valley locations using the non-parametric Wilcoxon ranksum test [Figure 1B(v)]. The resultant statistical value (*Z*-score) represents the magnitude of PAC, as mentioned before. Higher magnitudes of the *Z*-score indicate stronger PAC.

(6) $$\begin{array}{r} A_{\text{HF}}=gwf\;*\;a_{\text{HF}} \end{array}$$

Where *gwf* is Gaussian window function of length one-fourth of the cycle of the low-frequency value in pair.

An LF event (peak or valley) is necessary for PAC phenomenon; therefore, it is desirable to consider only LF events with higher amplitude (Aru et al., 2015). Moreover, an LF event with small amplitude is more likely to be affected by noise. Keeping these factors in view, we only considered the first *q*-percentile of peaks and *q*-percentiles of valley locations with higher magnitudes. Then, the distribution of the HF amplitude at peak and valley locations was statistically compared and the *Z*-score was computed. This was performed for *q* = 95, 75, 50, 25, and 10. We refer to this PAC computation method as “PvsV-HF(q)” in the subsequent text, where “*q*” symbolizes the *q*-percentile.

#### Correlation-based PAC (corrPAC)

We also computed amplitude-amplitude coupling (AAC) and PAC using the correlation-based approach. The AAC score (corrAAC) is obtained by computing the correlation between the HF amplitude time course [i.e., *A*_*HF*_(*t*)], and the absolute LF amplitude time course [i.e., |*Re*_*a*_LF_(*t*)|], considering only peak and valley time locations (Equation 7). Indeed, in an earlier study (Penny et al., 2008), PAC scores were derived by computing the correlation between the absolute HF time course and the real component of the LF time course. However, this process also includes underlying AAC that is inappropriate with regard to the PAC phenomenon (Özkurt and Schnitzler, 2011). Thus, we computed the correlation-based PAC score in two ways. Firstly, we computed corrPAC1 by computing the correlation coefficient between the LF amplitude time course [i.e., *Re*_*a*_LF_(*t*)], and the residual of the HF time course after regressing out the HF time course, [i.e., *A*_HF_(*t*)] from the absolute LF time course [i.e., |Re_*a*_LF_(*t*)|] (Equations 8 and 9). The regression step used here suppresses the underlying AAC. Second, we computed corrPAC2 by computing the correlation between the HF time course, i.e., *A*_HF_(*t*), and the LF time course after applying the sign function, [i.e., sign(Re_*a*_LF_(*t*))] (Equation 10). As the sign function replaces the peak and valley amplitudes with +1 and –1, respectively, no notion of AAC remains. Thus, both these measures capture the PAC that is the modulation of the HF amplitude along with the LF peak and valley events whereas they are robust to be influenced by the underlying AAC.

(7) $$\begin{array}{rcl} \text{corrAAC} & = & \text{corr}\left(A_{\text{HF}}\left(t\right),\;|\text{Re\_}a_{\text{LF}}\left(t\right)|\right),\;\\ t\;\in \left(\text{peak or valley}\right) \end{array}$$

(8) $$\begin{array}{rcl} \text{res\_ }A_{\text{HF}}\left(t\right) & = & \text{residuals}\left\{\text{regress}\left(A_{\text{HF}}\left(t\right),\;|\text{Re\_}a_{\text{LF}}\left(t\right)|\right)\right\},\\ t\;\in \left(\text{peak or valley}\right) \end{array}$$

(9) $$\begin{array}{rcl} \text{corrPAC}1 & = & \text{ corr}\left(\text{res\_ }A_{\text{HF}}\left(t\right),\text{ Re\_}a_{\text{LF}}\left(t\right)\right),\;\\ t\;\in \left(\text{peak or valley}\right) \end{array}$$

(10) $$\begin{array}{rcl} \text{corrPAC}2 & = & \text{ corr}A_{\text{HF}}\left(t\right),\text{ sign}\left(\text{Re\_}a_{\text{LF}}\left(t\right)\right),\;\\ t\;\in \left(\text{peak or valley}\right) \end{array}$$

#### Statistical evaluation of PAC score

For each elementary MEG source signal, we generated 500 surrogate signals using each kind of surrogate approach separately. After that, we computed the PAC score for the elementary MEG source signal from each node and corresponding different surrogate data using the different PAC computational methods separately (i.e., MI, PvsV-HF and corrPAC). From the bootstrapped distribution of PAC scores (500 surrogate iterations), the 99th quantile value (corresponding to *p* < 0.01) was determined as a threshold value (T_PAC_) for each kind of surrogate measure. A node is said to exhibit PAC phenomenon if the PAC score for the elementary source signal exceeds the threshold value (T_PAC_). The significance of PAC phenomenon for each of the nodes was evaluated separately for each of the surrogate measures, computational methods, and frequency pairs.

## Results

### Surrogate data and PAC

**Figure 2:**
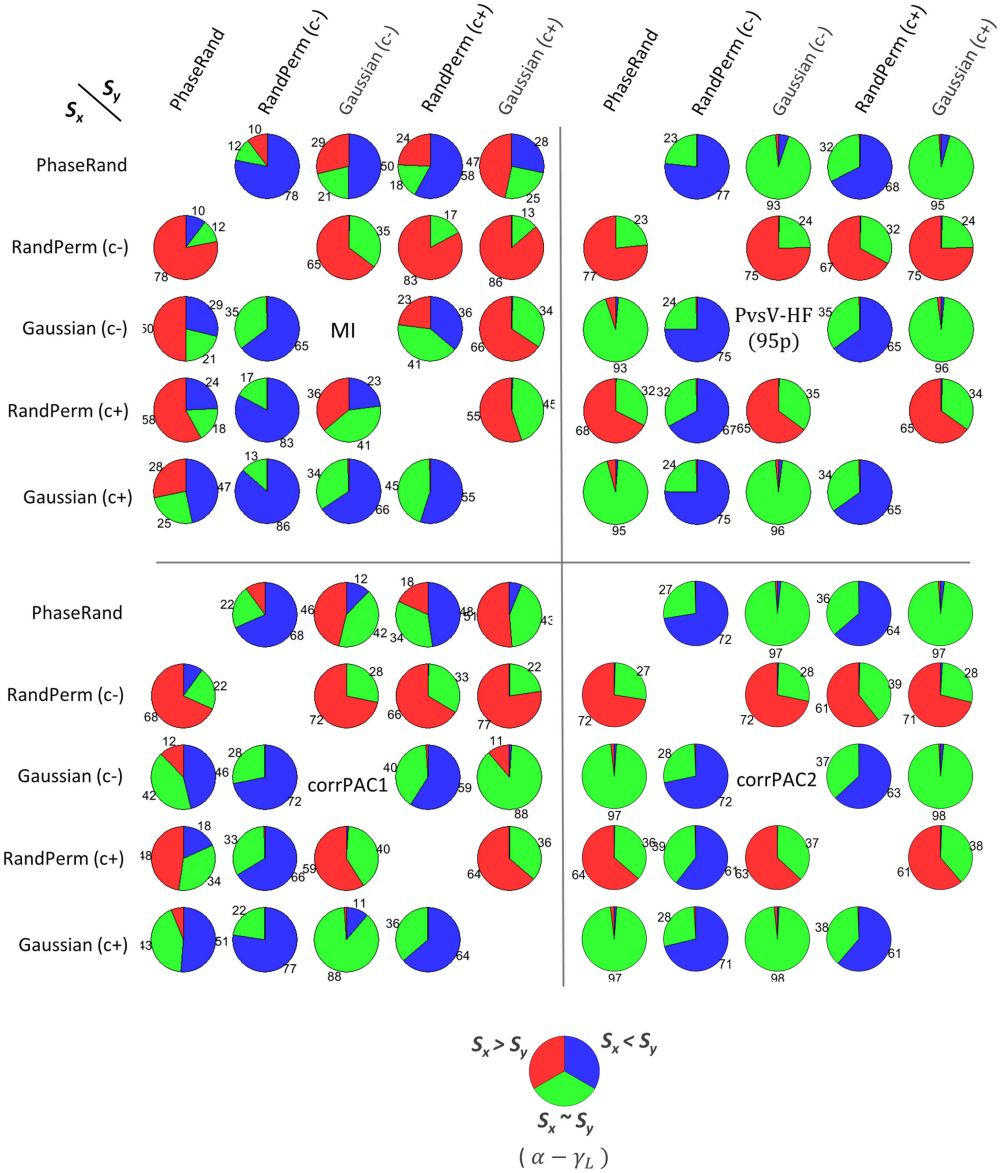
**Pairwise comparison of PAC scores among different surrogate measures (*S_x_*, *S_y_*) for frequency pair alpha-lower gamma (α − γ_*L*_), for different PAC computation methods**. (*S_x_* > *S_y_*, red color) or (*S_x_* < *S_y_*, blue color) represents the proportion of nodes (out of 58 nodes × 27 subjects = 1566 nodes) that had higher threshold PAC-value (T_PAC_, that is the 99th quantile value in surrogate PAC distribution) between two surrogate measures, and additionally showed significant (Wilcoxon rank-sum test, *p* < *0.01, n* = *500*) difference in distribution of PAC score between two surrogate measures. (*S*_*x*_ ~ *S_y_*, green color) indicates no difference (Wilcoxon rank-sum test, *p* > *0.01, n* = *500*).

We determined the threshold PAC-value (T_PAC_) that is the 99th quantile value of the PAC score in the surrogate PAC score distribution for each of the nodes, surrogate measures, and PAC computation methods. Then, we performed a pairwise comparison of surrogate PAC score distributions between various surrogate measures for each of the nodes. In a pairwise comparison, we identified the proportion of nodes (out of 58 nodes × 27 subjects = 1566 nodes) that had a higher threshold PAC-value (T_PAC_) for a particular surrogate measure compared to another surrogate measure. The probability of two threshold PAC scores determined from two different surrogate measures being equal is the lowest. Therefore, we applied an additional conditionality that there should be a significant difference (Wilcoxon rank-sum test, *p* < 0.01, *n* = 500) in the surrogate PAC score distribution between these two surrogate measures. Otherwise, these should be considered equal. Collectively, these results provide an idea about the tendency of a particular surrogate measure to produce higher PAC scores, which indicates the conservativeness of a surrogate measure. For example, the pie chart in the first row and second column in Figure 2 [*S*_*x*_: PhaseRand; *S*_*y*_: RandPerm(c–); MI method] is interpreted as follows: about 78% of nodes [*S*_*x*_: PhaseRand < *S*_*y*_: RandPerm(c–); blue color] showed higher threshold PAC-values (T_PAC_) for the RandPerm(c–) surrogate measure than the PhaseRand surrogate measure, and there was a significant difference in the surrogate PAC score distributions between them. About 10% of nodes [*S*_*x*_: PhaseRand > *S*_*y*_: RandPerm(c–); red color] showed higher threshold PAC-values (T_PAC_) for the PhaseRand surrogate measure than the RandPerm(c–) surrogate measure, and there was a significant difference in the surrogate PAC score distributions between them. However, about 12% of nodes [*S*_*x*_: PhaseRand ~ *S*_*y*_: RandPerm(c–); green color] did not show a significant difference in surrogate PAC score distributions. Overall, the RandPerm(c–) surrogate measure produced higher threshold values (i.e., more conservative) than PhaseRand surrogate measure. Considering all such pairwise comparisons among the surrogate measures (Figure 2), RandPerm, in comparison to the PhaseRand and Gaussian surrogate measures, showed higher threshold values (i.e., more conservative) irrespective of the PAC computational method. Moreover, surrogate measures without coloring [i.e., Randeperm(c–) and Gaussian(c–)] showed higher threshold values compared to surrogate measures with coloring [i.e., RandPerm(c+) and Gaussian(c+)]. In the case of the PvsV-HF and corrPAC2 PAC computation methods, the majority of nodes (more than 90%) did not show significant differences in PAC score distributions among PhaseRand, Gaussian(c+), and Gaussian(c–) surrogate measures. Overall, as expected, the significance of the PAC phenomenon depends on the surrogate measures and PAC computation methods that were used for evaluation.

**Figure 3:**
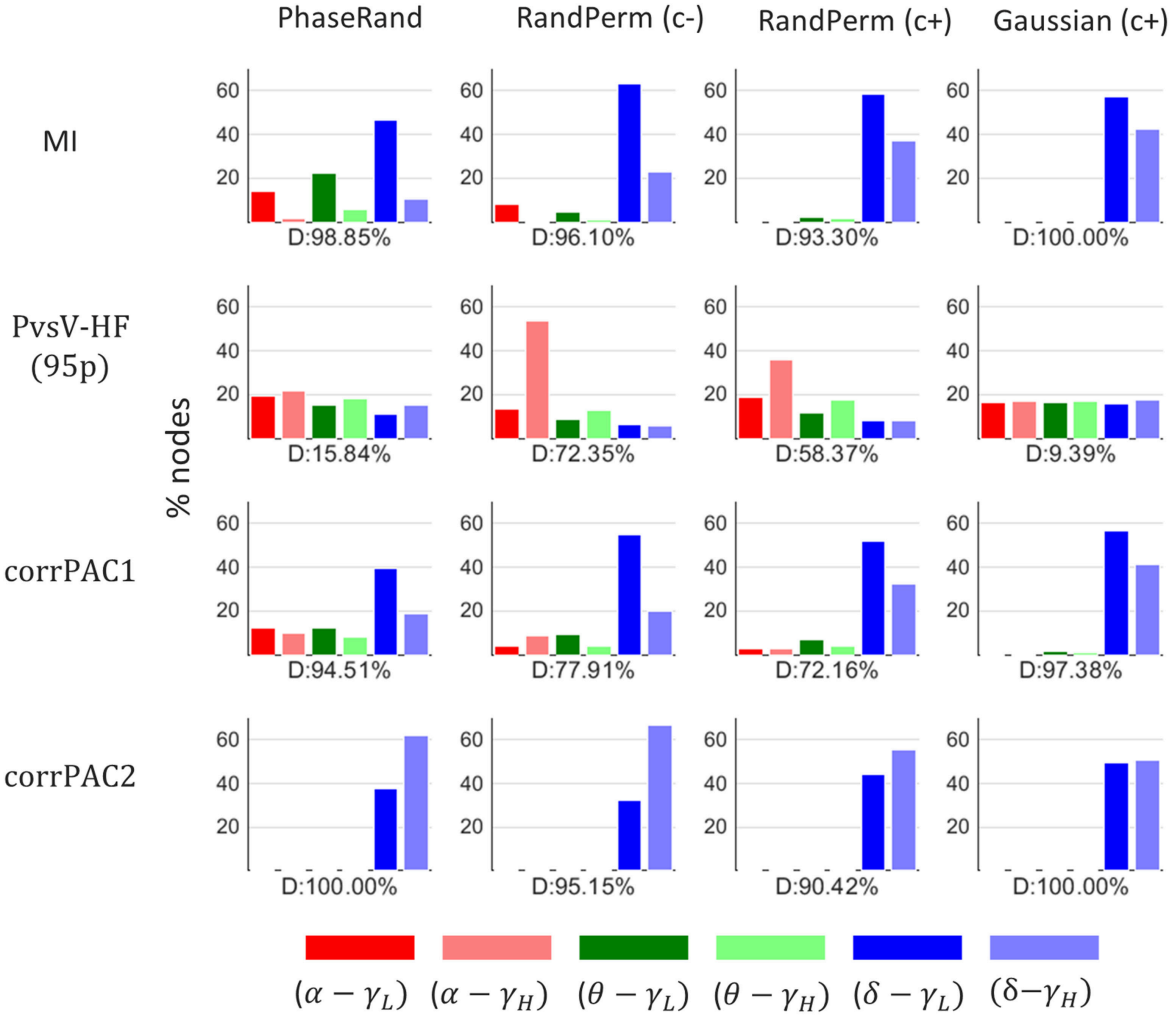
**Proportion of nodes (percentage out of 58 nodes × 27 subjects = 1566 nodes) that showed higher threshold PAC-value for a particular frequency pair after a direct comparison amongst frequency pairs**. D, proportion (%) of nodes that showed a significant difference in PAC score distribution amongst frequency pairs (*Friedman test, p* < *0.01*).

We performed a direct comparison of surrogate PAC score distributions among frequency pairs for each of the nodes, surrogate measures, and PAC computation methods. A major proportion (40–100%) of nodes (% out of 58 nodes × 27 subjects = 1566 nodes) showed a significant difference (Friedman test, *p* < 0.01, *n* = 500) in surrogate PAC score distributions among frequency pairs for each of the surrogate measures and PAC computation methods except for the PvsV-HF(95) PAC computation method with PhaseRand and Gaussian surrogate measures (D, Figure 3). In addition, the proportion of nodes (percentage out of 58 nodes × 27 subjects) that showed the highest threshold values (T_PAC_) for a particular frequency pair upon a direct comparison among frequency pairs is depicted in Figure 3 (color bar). The PAC score computed through MI and corrPAC methods showed a high proportion of nodes that had higher threshold values (T_PAC_) for frequency pairs with delta frequency than with theta and alpha frequencies irrespective of the surrogate measure used. As these results indicate that magnitudes of PAC score vary across different frequency pairs for the same surrogate data, we should avoid a direct comparison between PAC score distributions or combine PAC score distributions across different frequency pairs. Therefore, first, we should evaluate the significance of the PAC score within a frequency pair and then we can do comparative analysis between the various frequency pairs.

### Resting-state PAC

**Figure 4:**
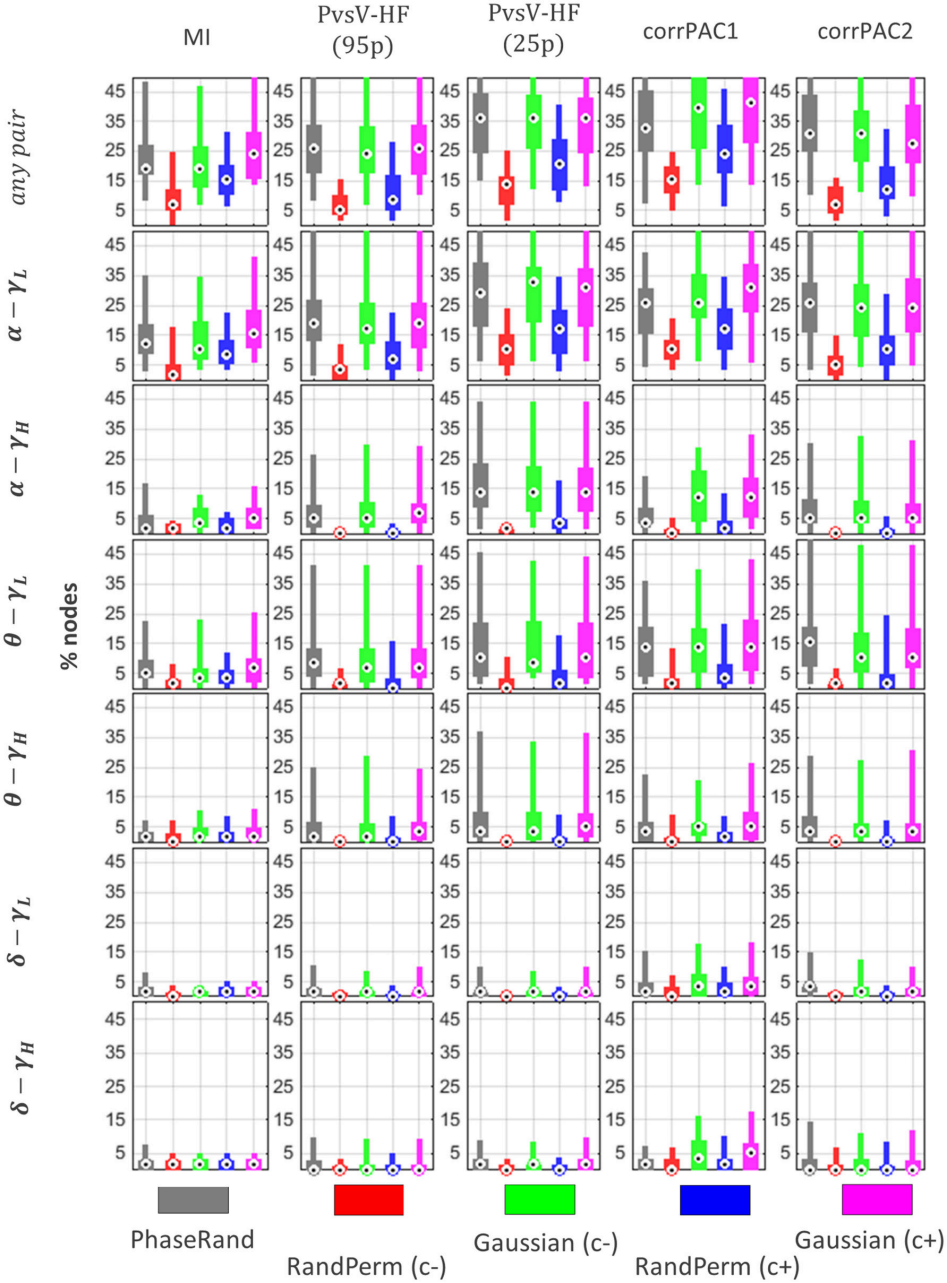
**Resting state PAC phenomenon in MEG source imaging**. Distribution of proportion of nodes (percentage out of 58 nodes) across the subjects that showed significant PAC phenomenon. Here, “any pair” represents significant PAC in any one or more frequency pairs. Significance was evaluated using different surrogate measures i.e., PhaseRand, RandPerm(c–), Gaussian(c–), RandPerm(c+), and Gaussian(c+), and using different PAC computation methods i.e., MI, PvsV-HF(p95), PvsV-HF(p25), corrPAC1, and corrPAC2.

The PAC score from a resting-state MEG source signal for a particular node is significant if it exceeds the threshold value (T_PAC_) that is the 99th quantile (*p* < 0.01) value in the surrogate PAC score distribution (500 iterations). The distribution of proportions of nodes (percentage out of 58 nodes) across the subjects that showed significant PAC scores for various surrogate measures, frequency pairs, and PAC computation methods is illustrated in Figure 4. There was a significant difference (Friedman test, *p* < 0.01, *n* = 27) in the distribution of proportions of nodes with significant resting-state PAC phenomenon across the subjects among different surrogate measures (in any frequency pairs, Figure 4[any pair]). In *post-hoc* analysis (pairwise Wilcoxon signed-rank tests, *p* < 0.01; Bonferroni correction applied, *n* = 27), the smallest proportion of nodes with resting-state PAC was seen for the RandPerm(c–) surrogate measure, whereas the highest proportion of nodes with resting-state PAC was seen for the Gaussian(c+) surrogate measure when evaluated with the MI and corrPAC1 methods. However, there was no significant difference in the outcome using the PhaseRand, Gaussian(c–), and Gaussian(c+) surrogate measures when evaluated using the PvsV-HF and corrPAC2 methods. Considering the least conservative surrogate measures [any frequency pair, Gaussian(c+), Figure 4], about 25% of nodes for the MI method, 36% of nodes for the PvsV-HF(25) method, and 41% of nodes for the corrPAC1 method showed significant resting-state PAC phenomenon. Considering the most conservative surrogate measures [any frequency pair, RandPerm(c–), Figure 4], about 7% of nodes for the MI method, 13% of nodes for the PvsV-HF (25) method, and 16% of nodes for the corrPAC1 method showed significant resting-state PAC. While comparing outcomes from frequency pairs, primarily the alpha-lower gamma (α−γ_*L*_) frequency pair showed a higher proportion of nodes with resting-state PAC phenomenon, whereas the extent of the resting-state PAC phenomenon was comparatively low or nearly absent in other frequency pairs (Figure 4). There was a significant difference (Friedman test, *p* < 0.01, *n* = 27) in the outcome between PAC computational methods for a given surrogate measure. In *post-hoc* analysis (pairwise Wilcoxon signed-rank tests, *p* < 0.01; Bonferroni correction applied, *n* = 27), the MI method showed the lowest proportion of nodes, whereas the corrPAC1 method showed the highest proportion of nodes with significant resting-state PAC phenomenon. In regards to LF events with higher amplitudes, there was a significant increase (about 7–10%) in the number of nodes with resting-state PAC phenomenon (PvsV-HF method, Figure 4; Supplementary Figure S1). Moreover, surrogate measures showed more impact on the extent of resting-state PAC compared to PAC computational methods. In node-specific analysis across the subjects (Supplementary Figure S3), none of the nodes showed significant resting state PAC phenomenon in more than 60% of subjects (<50% subjects for the majority of nodes) even using the least conservative surrogate measure and PAC computation method.

### Dynamic resting-state PAC

**Figure 5:**
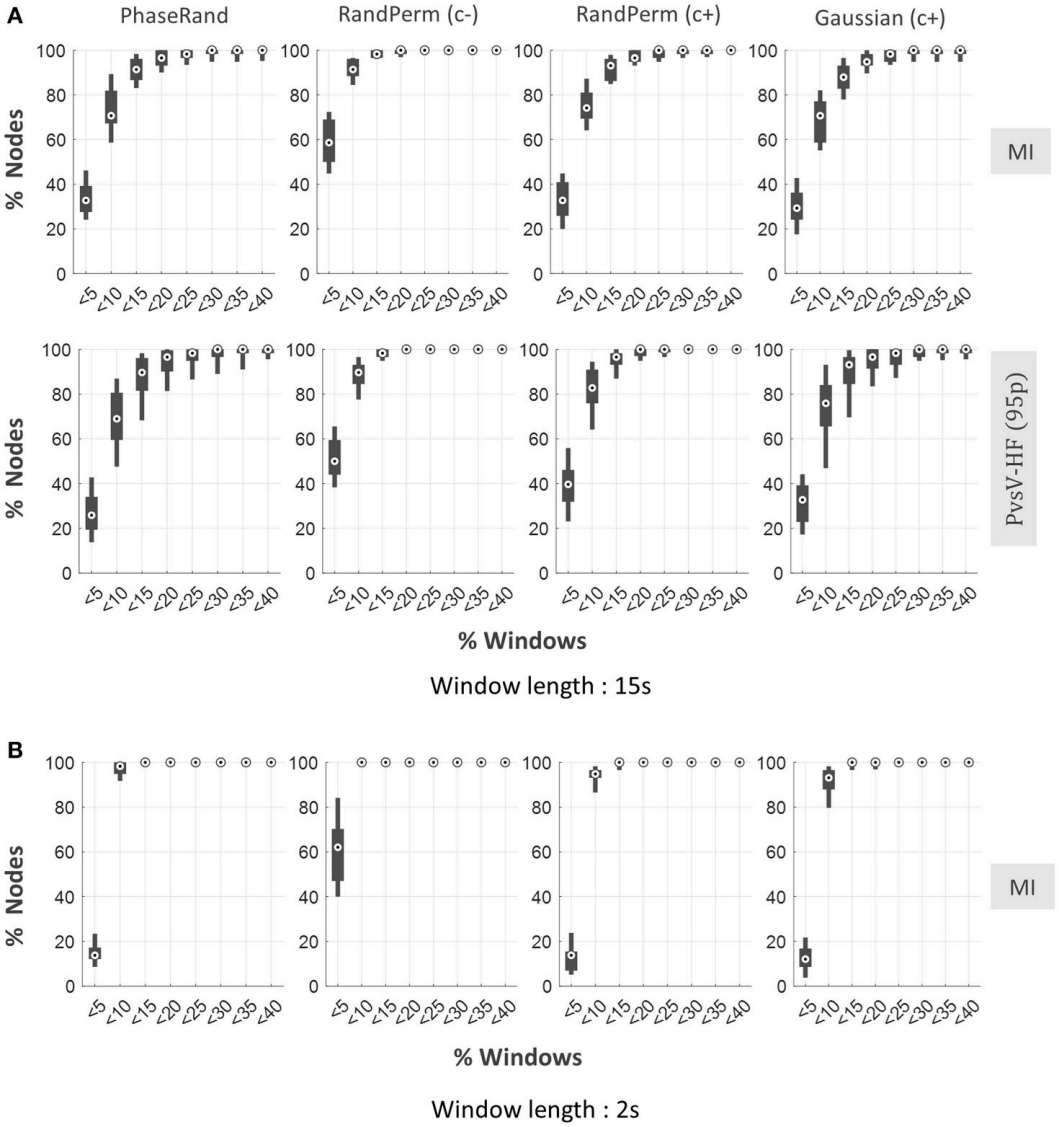
**Dynamic resting state PAC phenomenon in MEG source imaging**. Distribution of proportion of nodes (out of 58 nodes) across the subjects against the proportion of windows with significant PAC in any one or more frequency pairs, for PhaseRand, RandPerm(c–), RandPerm(c+) and Gaussian(c+) surrogate measures, and for MI and PvsV-HF(95) PAC computation methods **(A)** for window time length 15 s, **(B)** for window time length 2 s.

We evaluated resting-state PAC phenomenon over multiple windows (time segments) with short durations from continuous resting-state MEG signals. Here, we considered window lengths of 15 and 2 s. Continuous resting state MEG source signals were segmented into multiple windows of 15 or 2 s with an overlap of 7.5 or 1 s, respectively, between two successive windows. The PAC score for each window was computed and evaluated similarly to the continuous signal described above. For shorter signals, it is only feasible to use the MI (for 2 s window) or MI and PvsV-HF(95p) (for 15 s window) PAC computation methods to measure PAC scores. For a window length of 15 s, on average about 70% (median) and 90% (median) of nodes showed <10% of windows to have significant PAC when evaluated with the least and more conservative surrogate measures, respectively (Figure 5). However, the majority of nodes (more than 95%) showed <20% of windows as having significant PAC scores even when they were evaluated using the least conservative surrogate measure (Figure 5). However, the proportion of windows with significant PAC further dropped for a window length of 2 s. In this case, the majority of nodes (more than 95%) showed <10% of windows to have significant PAC scores even when evaluated with the least conservative surrogate measure (Figure 5). In the node-specific analysis, none of the nodes showed more than 10% of windows to have significant PAC scores in more than 60% of subjects even when the least conservative surrogate measure was used (Supplementary Figure S4). Overall, the results indicate that resting-state MEG signals failed to exhibit ubiquitous PAC phenomenon over multiple time segments even when evaluated using the least conservative surrogate measure.

## Discussion

In the present work, we analyzed the within a node PAC phenomenon in the resting-state brain using MEG source imaging modality. We also investigated the impact of different surrogate measures and PAC computational methods on the extent of PAC phenomenon. Our results indicate that the extent of PAC phenomenon mainly depends on surrogate measures, PAC computational methods, and evaluation approaches. Therefore, the approach to PAC phenomenon evaluation is critical for drawing conclusions about the outcome.

Surrogate data generated through the shuffling of either the LF phase or the HF amplitude time course is frequently used for PAC evaluation (Penny et al., 2008; Özkurt and Schnitzler, 2011; van der Knaap and van der Ham, 2011; Dvorak and Fenton, 2014). PhaseRand surrogate measure used in the present study (by introducing a minimum shuffling) destroys specific cyclostationarity but maintains non-specific cyclostationarity and it was considered to be a more appropriate conservative measure (Aru et al., 2015). The PhaseRand and Gaussian surrogate measures showed a similar level of conservativeness. This similarity was present probably because MEG signals themselves are non-stationary or quasi-stationary. Consequently, the concept of cyclostationarity between the LF and HF components had no impact even after a minimum shuffling of either the amplitude or the phase time course, particularly for a long, continuous signal. In the present study, the RandPerm surrogate measure appeared to be the most conservative compared to Gaussian and PhaseRand surrogate measures. Data with an asymmetric distribution, for instance, with more positive or negative spikes, is likely generate false positive PAC. The RandPerm surrogate measure partly considers such asymmetry in the data distribution related to a non-physiological PAC. However, this is not the case for Gaussian surrogate data (that have a symmetric distribution) and PhaseRand surrogate measure (particularity for continuous signals). Nevertheless, we maintain the notion that none of the surrogate measures mentioned above are perfect and can completely capture the specific PAC, but they are robust to non-specific PAC. Indeed, the PhaseRand and RandPerm surrogate measures still take certain considerations of data properties that affect the PAC score. However, it is worth mentioning that the Gaussian surrogate measure hardly takes into account any knowledge of data related to a non-physiological PAC and, therefore, is the least suitable surrogate measure. Coloring of the signal (introducing 1/*f* spectral characteristic) lowers the PAC score value in surrogate data. Hence, it is less conservative than without coloring, because coloring the signal destroys all of the spikes or nonlinearity, but this is not the case for the elementary raw signal.

In the present study, we used three different kinds of PAC computation approaches, not with the aim to find the best one but to see their effects on PAC estimation. The MI computation method has frequently been used as a PAC measure in many prior studies. However, it does not consider the amplitude of LF events, and it is susceptible to HF outlier events (noisy spikes with high amplitude) as they directly contribute to the PAC score. In contrast, the PvsV-HF method considers the LF events with a higher amplitude that are essential for PAC phenomenon (Aru et al., 2015). Moreover, it is a statistical approach and less susceptible to HF outlier events (few noisy spikes with high amplitude), as they do not directly contribute to the PAC score. Correlation-based PAC scoring methods as proposed here provide another way to capture PAC phenomenon and are robust to be influenced by the underlying AAC. Moreover, they provide an identical way to compute AAC and PAC so that they can be directly compared. In contrast to the MI and PvsV-HF computation methods, correlation-based PAC estimation is mainly suitable for continuous signals with longer durations. Interestingly, the extent of PAC phenomenon, i.e., the proportion of nodes with significant PAC phenomenon, is almost similar for the MI and PvsV-HF(95p) PAC computational methods. However, the inclusion of LF events in the PvsV-HF method increases the extent of PAC phenomenon. Moreover, the corrPAC1 computational method produced a greater extent of PAC than the other alternatives used in the present study. Nevertheless, it is hard to assert a choice for one particular method over another for PAC estimation in longer-duration continuous signals.

We observed greater PAC phenomenon for alpha-lower gamma frequency pair (α−γ_*L*_), which is consistent with others studies on PAC phenomenon in the resting-state brain (Wibral et al., 2013; Berman et al., 2015). However, a spike (physiological or non-physiological) in the signal evokes a broadband frequency response. Therefore, closer values of LF and HF frequencies in frequency pairs are likely to contribute to the non-physiological PAC that results from the broadband response from spikes (Kramer et al., 2008). Thus, this could be a contributing factor to the observed high PAC scores for alpha-lower gamma frequency pair, and, consequently, for PAC phenomena in general. Many previous studies have analyzed PAC phenomenon using EcoG/LFP data and often focused on HF components higher than 80 Hz (Canolty and Knight, 2010; de Hemptinne et al., 2013; Voytek et al., 2013). In contrast, MEG signals have lower signal-to-noise ratios, particularity for such high-frequency oscillations (Muthukumaraswamy, 2013). Thus, this could be one of the factors for the observed minimal or absent PAC phenomenon in resting-state MEG data. However, it cannot be considered as the only contributing factor, as we can see ubiquitous AAC phenomenon even for frequency pairs with high gamma frequency (Supplementary Figure S2). In contrast to MEG, EcoG and LFP signals have higher signal-to-noise ratios for high frequency oscillations, but also have comparatively more positive or negative spikes that are susceptible to producing false positives.

The evaluation of resting-state MEG signals with the least conservative surrogate measure revealed limited extent of nodes with significant PAC phenomenon. On the other hand, an assessment with a more conservative approach showed that resting-state MEG signals failed to exhibit ubiquitous PAC phenomenon. We observed a greater extent of PAC phenomenon for frequency pairs containing alpha (10 Hz) compared to theta (6 Hz) and delta (3 Hz) LF-values. In contrast to the current outcome, a recent study on resting-state MEG signals (Florin and Baillet, 2015) showed that about half of voxels across the cortex exhibited significant PAC phenomenon, and, among them (60% of voxels), the maximum PAC phenomena occurred in a frequency pair with delta frequencies. In their method, the PAC score was measured using the MI computation method and colored Gaussian noise data that were used as a surrogate measure (similar to Gaussian(c+)) for significance evaluation. They determined the threshold PAC-value by considering the 95th quantile value in the surrogate distribution that was generated after combining all surrogate PAC scores from all frequency pairs. In the present study, we showed that a direct comparison or consideration of PAC scores across the frequency pairs is not appropriate, particularly for PAC scores computed using the MI method in which they are unsurprisingly higher for the delta frequencies irrespective of the surrogate measure. Therefore, the detected ubiquitous PAC phenomenon in resting-state MEG signals in the (Florin and Baillet, 2015) study is more likely a false positive outcome related to evaluation approach used, whereas the maximum PAC phenomenon in frequency pairs with delta frequencies is the incorrect conclusion.

Neural information processing in the brain is complex and flexible, and emerging views suggest that PAC phenomenon is dynamic over multiple time segments or epochs during the execution of sensory-motor tasks (Gupta and Chen, 2016). Similarly, dynamic functional connectivity analysis using MEG source imaging also reveals the appearance and disappearance of various sensory or sensory-motor networks over time in the resting brain (de Pasquale et al., 2010; Brookes et al., 2014). Moreover, recent studies have also suggested a possible role of ubiquitous PAC in assisting the resting state network (Wang et al., 2012; Weaver et al., 2016). However, in the present study, PAC phenomenon appeared to be very limited (for the 15 s window length) or almost absent (for the 2 s window length) over multiple time segments across the nodes (Figure 5). On the other hand, PAC phenomenon has often been observed in task-related activity for even shorter epochs in MEG/EEG imaging modalities (Demiralp et al., 2007; Papadaniil et al., 2015) or EcoG/LFP imaging modalities (Canolty et al., 2006; Voytek et al., 2013). For this discrepancy between resting-state and task conditions, we have two views. First, resting-state MEG signals have failed to reveal significant dynamic PAC phenomenon. Second, there is significant PAC phenomenon in post-stimulus epochs in task-related data. However, it may partially or entirely be comprised of false positive PAC phenomenon resulting from event-related potential spikes (or imperfect sinusoidal waveforms) in the post-stimulus epoch. PAC phenomenon evaluation in task-related epochs has frequently employed the “PhaseRand” surrogate measure (Voytek et al., 2013). In fact, classical methods that are used to capture PAC signatures are inherently ambiguous in differentiating between physiological PAC and non-physiological false positive PAC related to non-linear properties of an oscillatory signal (Aru et al., 2015). However, a using more conservative surrogate measure such as the “RandPerm,” can at least control such false positive PAC phenomenon to a certain extent.

In the present study, our focus was on the within a node PAC phenomenon where low frequency phase and high frequency amplitude components were taken from a single time series. Many researchers have often shown an interest in PAC phenomenon between nodes, where low frequency phase and high frequency amplitude components are taken from two different time series (nodes) (Wibral et al., 2013; Dimitriadis et al., 2016). PAC phenomenon between nodes has different interpretations and issues (Aru et al., 2015), and the findings and issues in the present study are not directly applicable to it. A recent MEG study (Florin and Baillet, 2015) suggested a synchronized gating hypothesis with respect to within a node PAC phenomenon as evaluated in the present study. Within a node PAC phenomenon essentially bridges the communication-through-coherence (Fries, 2005) and binding-by-synchronization hypotheses (Varela et al., 2001) in one proposition. However, in the present study, the resting-state MEG source signal did not show widespread and consistent within a node PAC phenomenon in any of the nodes in the brain. Therefore, it requires further evaluation using EcoG/LFP imaging modalities, and superior within a node PAC estimation method that is robust to non-physiological PAC.

In conclusion, resting-state MEG signals failed to exhibit ubiquitous PAC phenomenon. As PAC phenomenon is highly susceptible to non-physiological PAC, a more conservative approach is desirable to control false positive PAC. Moreover, PAC phenomenon involves the high frequency component of a signal, where MEG has a lower signal-to-noise ratio; we need to take great care to evaluate significant PAC.

## Ethics statement

We used online publicly available data recorded from human.

## Author contributions

BG conceived and designed the research, BG, SL, MK, KA, JK, HK, and KK performed analysis, prepared the figures and wrote the manuscripts. All authors approved the final version of the manuscript.

## Funding

This work was supported by the World Class Laboratory (WCL) program in Korea Research Institute of Standards and Science. Data were provided [in part] by the Human Connectome Project, WU-Minn Consortium (Principal Investigators: David Van Essen and Kamil Ugurbil; 1U54MH091657) funded by the 16 NIH Institutes and Centers that support the NIH Blueprint for Neuroscience Research; and by the McDonnell Center for Systems Neuroscience at Washington University.

### Conflict of interest statement

The authors declare that the research was conducted in the absence of any commercial or financial relationships that could be construed as a potential conflict of interest.

## Abbreviations

LF: Low frequency
HF: High frequency
CFC: Cross-Frequency Coupling
PAC: Phase-Amplitude Coupling
AAC: Amplitude-Amplitude Coupling.

**Surrogate measures:**
PhaseRand: Splitting either phase or amplitude time component of signal into two block at some random time point and then interchanged their positions
RandPerm (c–): Random permutation of original time series, (c–), Without coloring (introducing 1/f characteristics)
RandPerm (c+): Random permutation of original time series, (c+), With coloring (introducing 1/*f* characteristics)
Gaussian (c–): Gaussian time series whose length as of original signal, (c–), Without coloring (introducing 1/*f* characteristics)
Gaussian (c+): Gaussian time series whose length as of original signal, (c+), With coloring (introducing 1/*f* characteristics)

**Computational methods:**
MI: Modulation Index
PvsV-HF: Peak vs. Valley High Frequency amplitude
corrPAC: Correlation based Phase-Amplitude Coupling
corrAAC: Correlation based Amplitude-Amplitude Coupling.
