# Supplementary material for: Evaluation of Phase-Amplitude Coupling in Resting State Magnetoencephalographic Signals: Effect of Surrogates and Evaluation Approach
Source: Front Comput Neurosci. 2016 Nov 25;10:120. doi: 10.3389/fncom.2016.00120 (PMC5122594; doi:10.3389/fncom.2016.00120)
Supplement: Supplementary file 1 [file Presentation1.PDF]

frontiers

Supplementary Material

Evaluation of Phase-Amplitude Coupling in Resting State Magnetoencephalographic Signals: Effect of Surrogates and Evaluation Approach

Bakul Gohel, Sanghyun Lim, Min-Young Kim, Kyung-min An, Ji-Eun Kim, Hyukchan Kwon, Kiwoong Kim***

Center for biosignals, Korea Research Institute of Standards and Science (KRISS), Daejeon, Republic of Korea

***Correspondence to**:

Kiwoong Kim, Email: kwkim@kriss.re.kr

**** Co-correspondence to**:

Bakul Gohel, Email: dr.bakul.research@gmail.com

Text. A: Information of subjects that were used for analysis

As mentioned in the main text (section 2.1), we used resting state MEG data from 27 subjects out of 61 subjects publically available in Human Connectome Project (HCP) web portal (http://www.humanconnectomeproject.org/). From each subject, we used data from only one session out of three sessions.

Subject ID (Resting session number) are as follow.

100307(1), 108323(2), 113922(2), 133019(3), 146129(2), 156334(1), 162026(2), 166438(3), 175540(3), 181232(1), 185442(1), 189349(2), 191841(3), 204521(3), 205119(2), 221319(1), 250427(3), 293748(1), 352132(1), 352738(1), 559053(3), 568963(1), 660951(3), 662551(2), 665254(1), 680957(1), 877168(2).

Text. B: Information on node locations used for analysis

As mentioned in the main text (section 2.1), we used 58 nodes across the cortex for analysis. For the node selection, first cortex was partitioned into 23 partitions in each hemisphere according to Brodmann atlas, where, some Brodmann areas were merged to form single partition considering function and size (Fig. 1a). After that, one or two nodes (voxels) from each of partitions were selected manually for analysis. In total, we selected 58 nodes for analysis. Color code in Fig. 1a represents the partition while black dot represents the location of nodes.

Partition	Brodmann area	Number of node(s) selected from partition
1	17,18	1
2	19	2
3	5,7	2
4	39	1
5	40	1
6	1,2,3	2
7	4	2
8	6	2
9	8	1
10	9	1
11	10	1
12	46	1

Partition	Brodmann area	Number of node(s) selected from partition
13	44,45	1
14	47,11,12	1
15	41,42	1
16	22	2
17	21	1
18	20	1
19	38	1
20	37	1
21	32	1
22	24	1
23	23,31	1

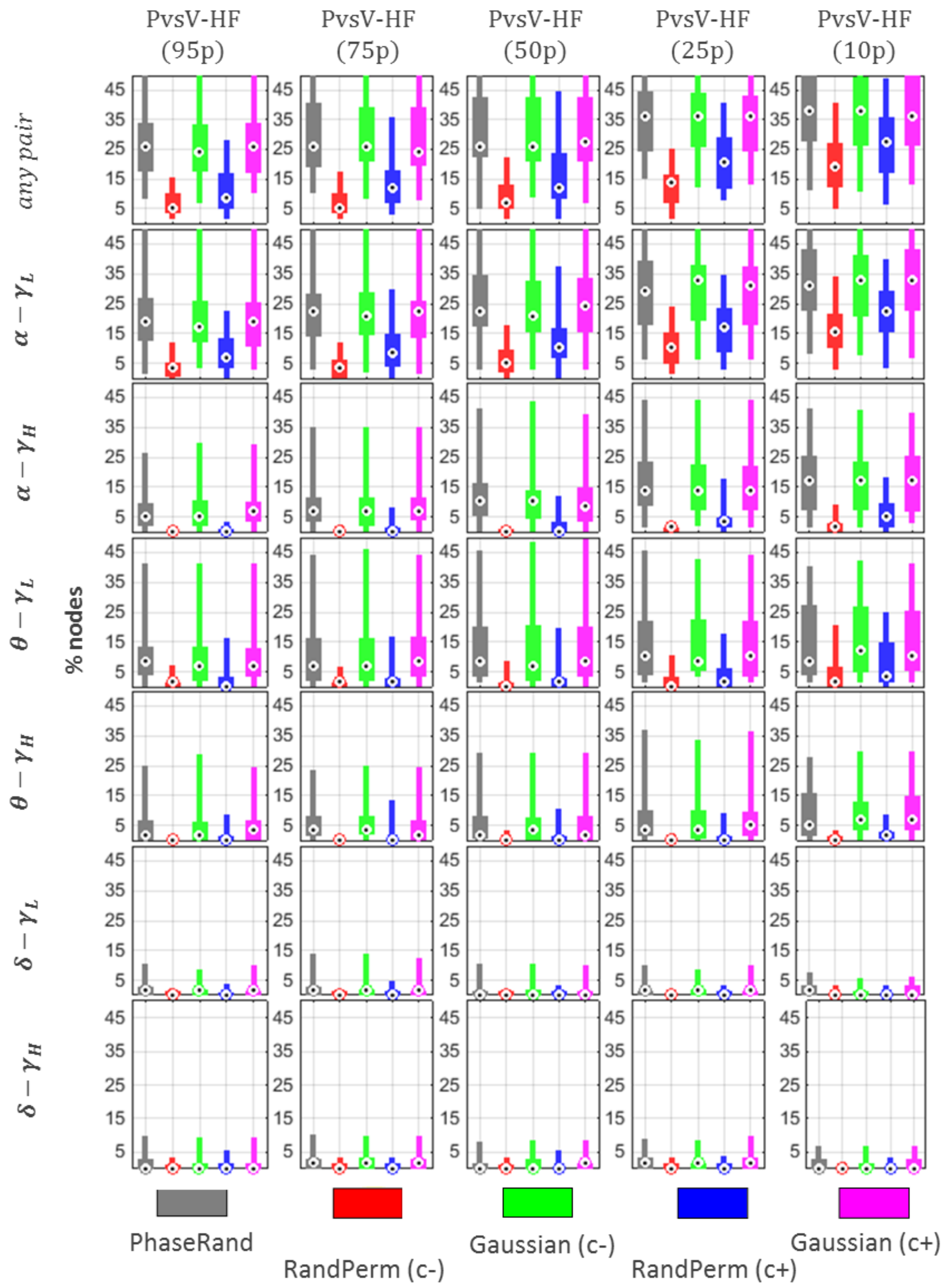

Supplementary Figure. S1. Distribution of proportion of nodes (% out of 58 nodes) across the subjects that show significant PAC phenomenon during resting state in different frequency pairs. "any pair" represents number of nodes with significant PAC in any one or more frequency pairs. Significance was evaluated using different surrogate measures i.e. PhaseRand, Randperm(c-),

Gaussian(c-), Randperm(c+) and Gaussian(c+), and PAC computation methods i.e. PvsV-HF(p95), PvsV-HF(p75), PvsV-HF(p50), PvsV-HF(p25), and PvsV-HF(p10).

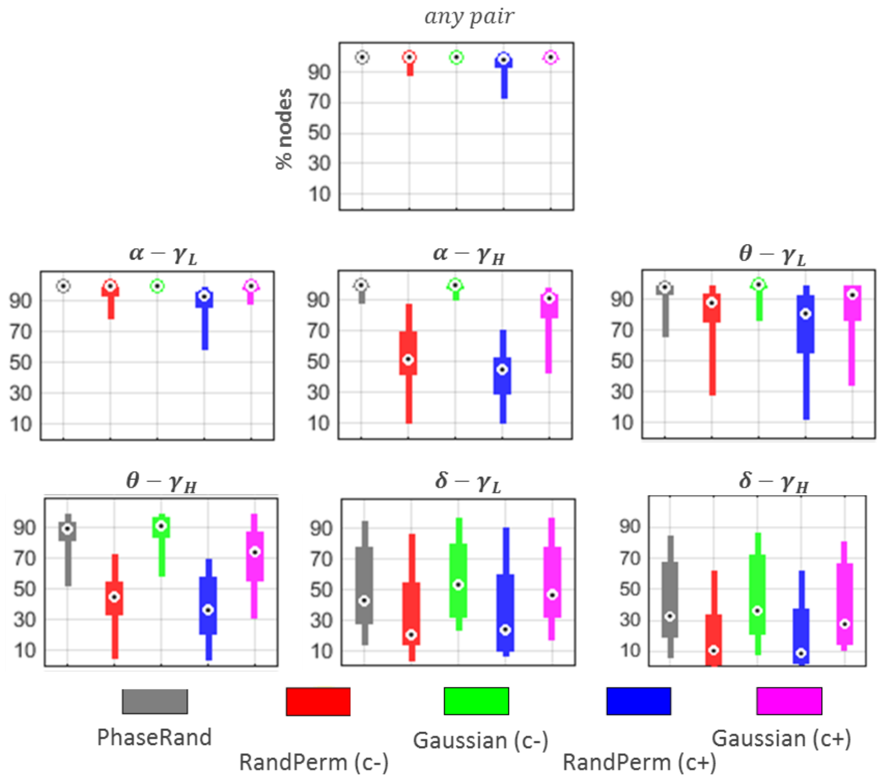

Supplementary Figure. S2. Distribution of proportion of nodes (% out of 58 nodes) across the subjects that show significant amplitude-amplitude coupling (AAC) during resting state in different frequency pairs. "any pair" represents number of nodes with significant PAC in any one or more frequency pairs. Significance was evaluated using different surrogate measures i.e. PhaseRand, Randperm(c-), Gaussian(c-), Randperm(c+) and Gaussian(c+).

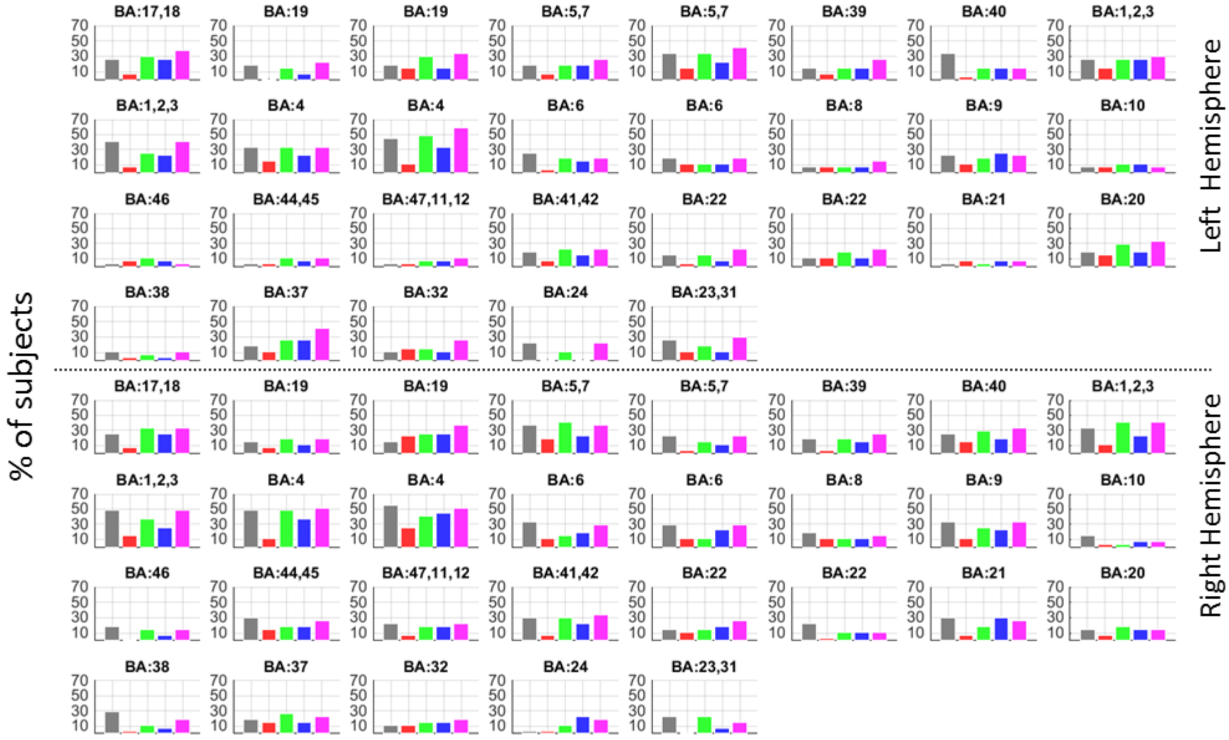

Figure.S3 (a) MI

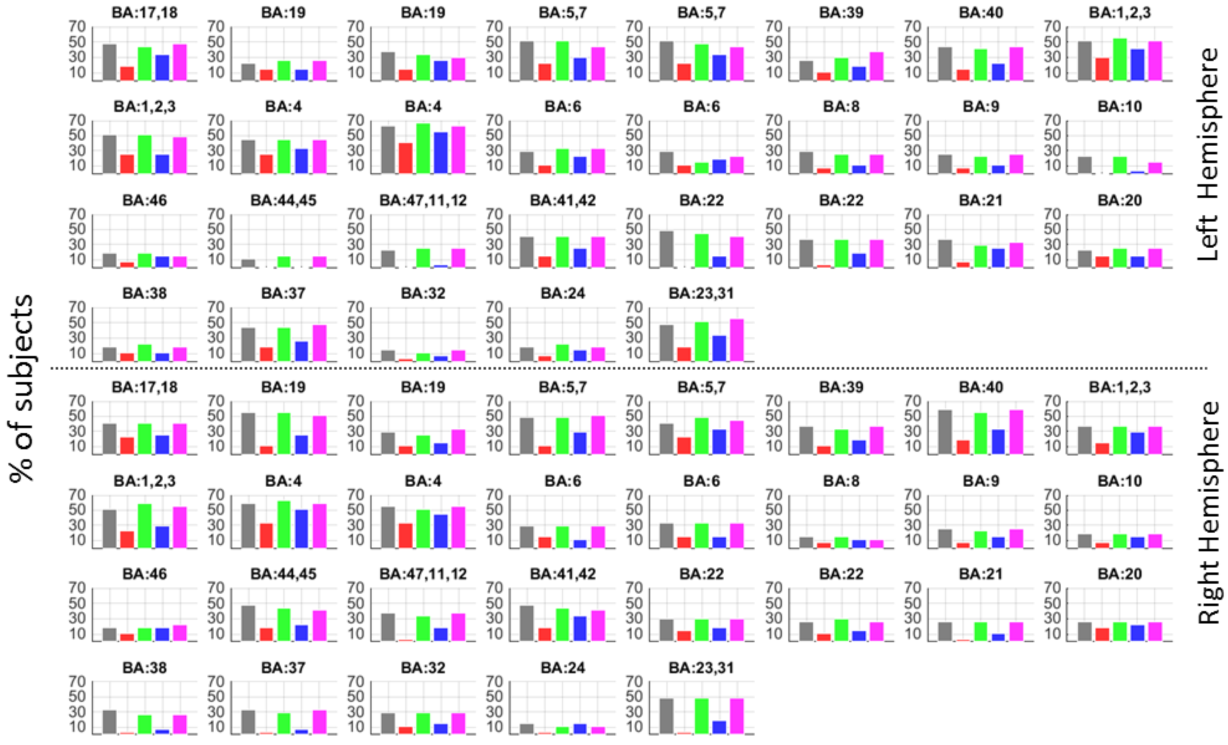

Figure.S3 (b) PvsV-HF(25p)

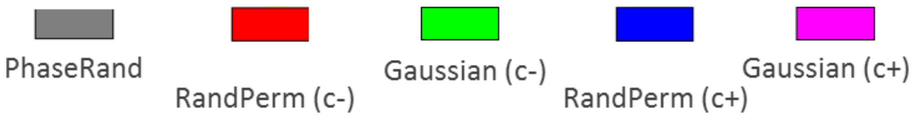

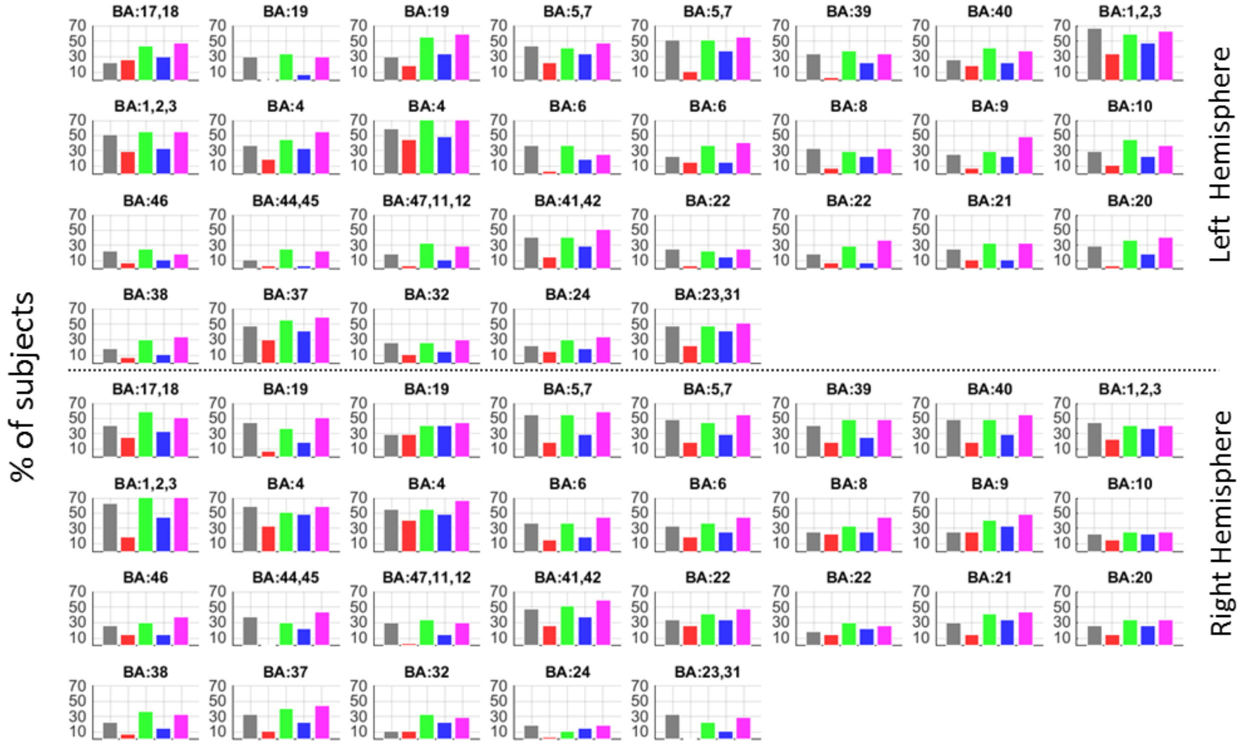

Figure.S3 (c) CorrPAC1

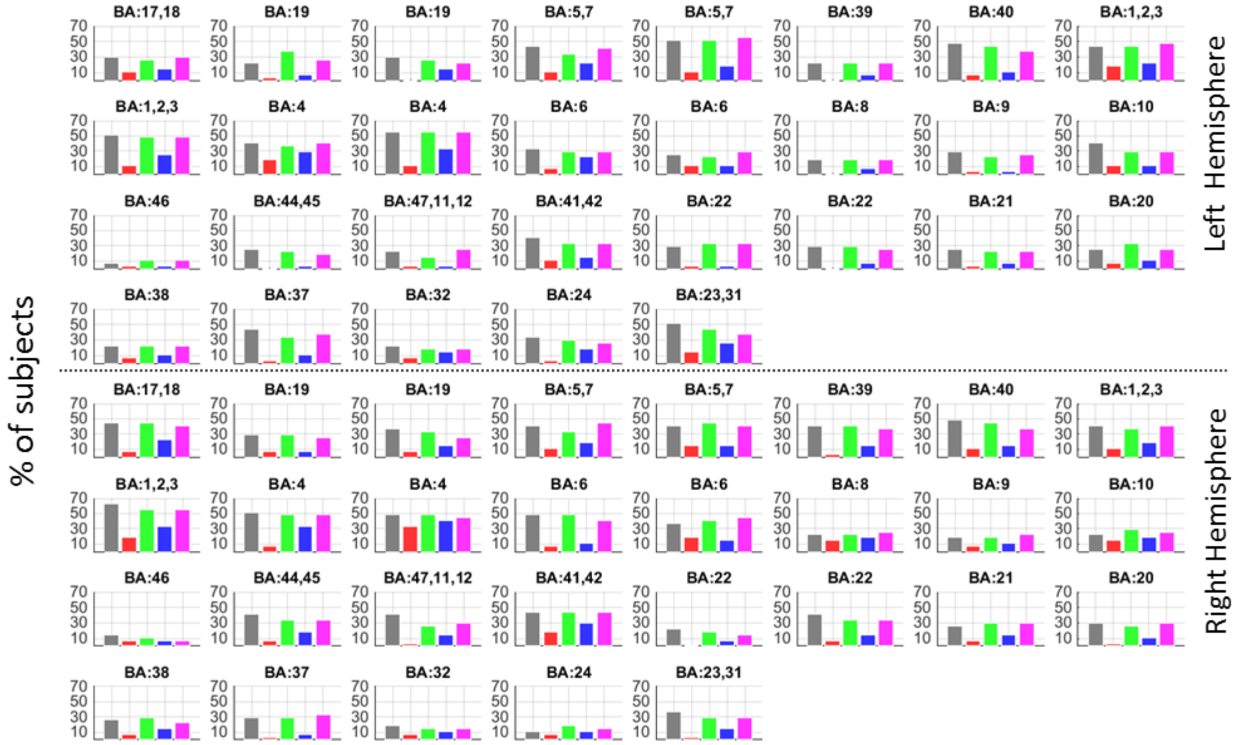

Figure.S3 (d) CorrPAC2

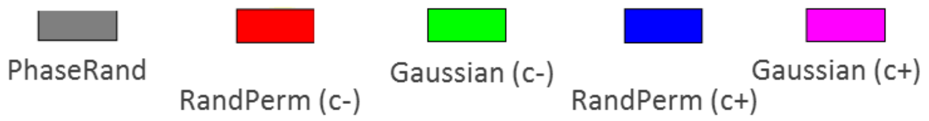

Supplementary Figure. S3. Node specific analysis. % of subjects with significant PAC (in one or more frequency pairs) for a given node. Significance evaluated using different surrogate measures i.e. PhaseRand, Randperm(c-), Gaussian(c-), Randperm(c+) and Gaussian(c+), and PAC computation method i.e. MI, PvsV-HF(25p), CorrPAC1 and CorrPAC2. BA: Brodmann area cluster, i.e. the location of the node.

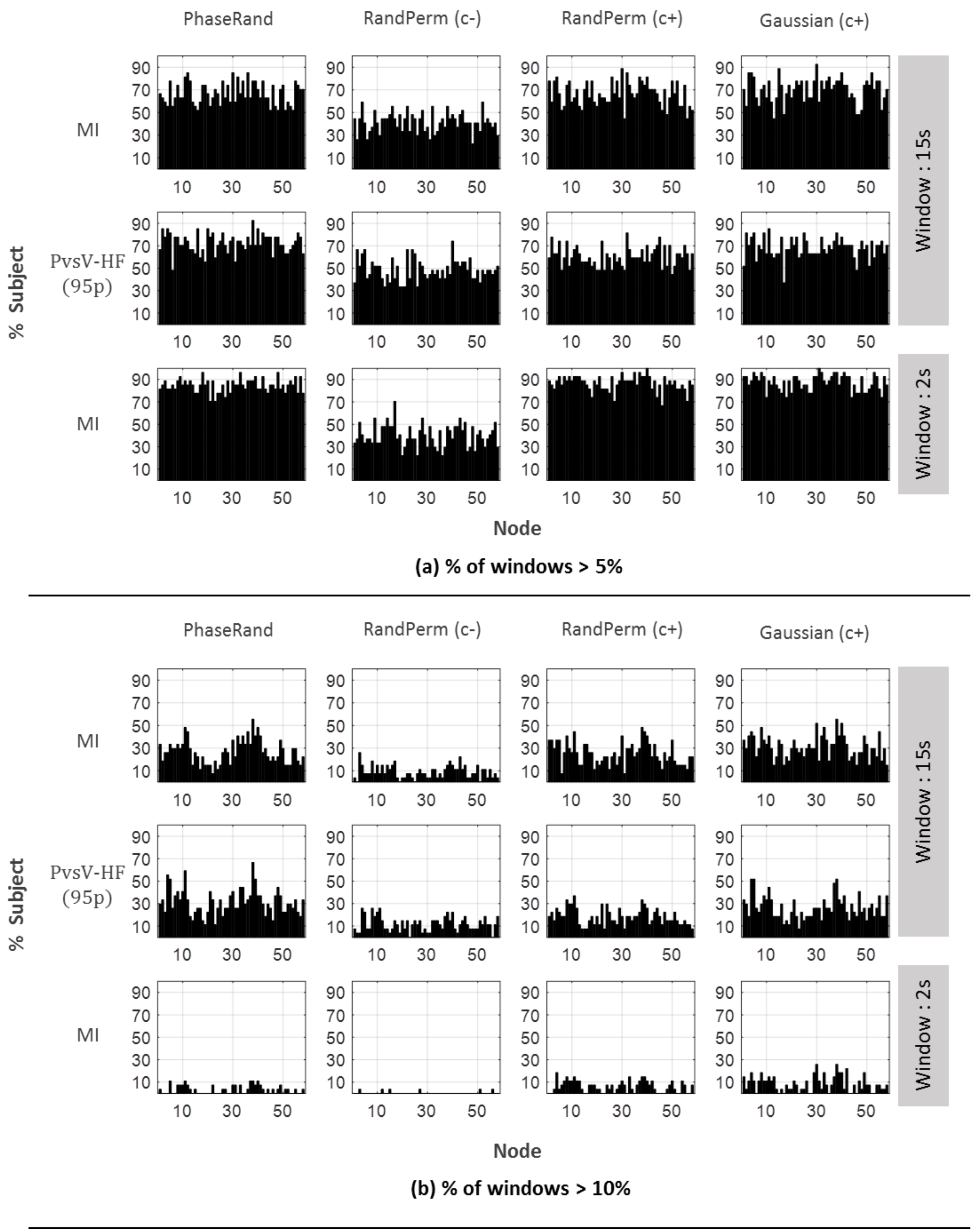

Supplementary Figure. S4. Node specific analysis for dynamic PAC. % of subjects with significant PAC(in one or more frequency pairs) in more than 5% (a) and more than 10% (b) windows for a given node(x-axis). Significance evaluated using different surrogate measures i.e. PhaseRand,

Randperm(c-), Randperm(c+) and Guassian(c+), and PAC computation methods i.e. MI and PvsV-HF(p25), for window length of 15s and 2s.
